# Evolutionary implications of new Postopsyllidiidae from mid-Cretaceous amber from Myanmar and sternorrhynchan nymphal conservatism

**DOI:** 10.1038/s41598-022-20897-y

**Published:** 2022-09-30

**Authors:** Jowita Drohojowska, Marzena Zmarzły, Jacek Szwedo

**Affiliations:** 1 Institute of Biology, Biotechnology and Environmental Protection, University of Silesia, 9, Bankowa Street, 40-007 Katowice, Poland; 2 Laboratory of Evolutionary Entomology and Museum of Amber Inclusions, Department of Invertebrate Zoology and Parasitology, University of Gdańsk, 59, Wita Stwosza Street, 80-308 Gdańsk, Poland

**Keywords:** Zoology, Evolution, Palaeontology, Phylogenetics, Taxonomy, Palaeoecology

## Abstract

Nymphs of extinct sternorrhynchan hemipterans are extremely rare, although very important for understanding of evolutionary traits of these insects. A protopsyllidioid nymph, in mid-Cretaceous amber from Kachin, Myanmar, placed in the family Postopsyllidiidae, is the first nymph of this family to be found in the fossil. Postopsyllidiidae previously comprised the sole genus *Postopsyllidium* with a few species: *P. rebeccae*, *P. grimaldii* and *P. burmaticum* from Kachin amber (Cenomanian) and *P. emilyae* from Turonian amber of New Jersey. Here, we report a new genus and species of postopsyllid *Megalophthallidion burmapteron* gen. et sp. nov. (imago) and the first known nymph of the family ascribed to the same genus. An overview of the fossil record of sternorrhynchan nymphs, and the importance of this finding, unlocking a new and complementary window to understanding the evolutionary traits of Protopsyllidioidea and other Sternorrhyncha hemipterans are presented.

## Introduction

Plant-sucking hemipterans of the suborder Sternorrhyncha Amyot et Audinet-Serville are cryptic plant parasites that live by eating phloem sap. With about 19,000 extant species, these insects are pale in comparison to the taxonomic diversity of some other plant‐feeding insect groups, but what makes them of special interest is their biology, in which they display extraordinary diversity. Almost all of them are tiny, sessile, and closely associated with their host plants. Phloem‐sap‐feeding damages plants, and sternorrhynchan species are among the worst agricultural pests. They affect plants directly through the loss of sap, but this is often outweighed by the damage they cause indirectly by facilitating microbial infections^1^. Sternorrhyncha have been evolving and diversifying for at least 290 million years, with the oldest known representatives in the Sakmarian/Artinskian of the early Permian.^2–7^. The nomenclature, classification, and relationships within the Sternorrhyncha are still subjects of discussions^8–14^. These discussions also concern extinct taxa currently placed in the superfamily Protopsyllidioidea Carpenter, 1931, comprising four families: Protopsyllidiidae Carpenter, 1931; Permopsyllidiidae Becker-Migdisova, 1985; Postopsyllidiidae Hakim, Azar et Huang, 2019; Paraprotopsyllidiidae Hakim, Azar, Szwedo, Drohojowska et Huang, 2021^6,7^. The family Postopsyllidiidae currently comprises the single genus *Postopsyllidium* Grimaldi, 2003 with 4 species, viz., 3 from early Cenomanian Burmese amber (*P. rebeccae* Grimaldi, 2003, *P. grimaldii* Hakim, Azar et Huang, 2019 and *P. burmaticum* Hakim, Azar et Huang, 2019), and a single species from Turonian New Jersey amber (*P. emilyae* Grimaldi, 2003). Records of fossil nymphs of Sternorrhyncha are very scarce; however, several hypotheses about their appearance and lifestyle have been proposed^15^. Therefore, finding another nymph, and the first one of the recently established family Postopsyllidiidae provides new insight into the evolutionary history of the group.

*Megalophthallidion burmapateron* gen. et sp. nov., established herein, is the second genus of Postopsyllidiidae and the first record of an imago and its corresponding nymph as amber inclusions. It possesses characters typical for Postopsyllidiidae, but also has unique, apomorphic features within Mesozoic postopsylloids; the nymph described here is the first known immature of Postopsyllidiidae.

## Results

### Systematic palaeontology

Order Hemiptera Linnaeus, 1758

Suborder Sternorrhyncha Amyot et Audinet-Serville, 1843

Superfamily Protopsyllidioidea Carpenter, 1931

Family Postopsyllidiidae Hakim, Azar et Huang, 2019

Genus *Megalophthallidion* Drohojowska et Szwedo, gen. nov.

LSID urn:lsid:zoobank.org:act:A6F71390-9B8E-4A19-8F30-C2A024B6EFB1

#### Type species

*Megalophthallidion burmapateron* Drohojowska et Szwedo, sp. nov.; by present designation and monotypy.

#### Etymology

Generic name is derived from Classic Greek megas (μέγας)—large, ophthalmos (ὀφθαλμός)—an eye and Greek form of generic name *Psyllidium*. Gender: masculine.

#### Type locality

Northern Myanmar: state of Kachin, Noije bum 2001 Summit Site amber mine in the Hukawng Valley, SW of Maingkhwan.

#### Type stratum

Lowermost Cenomanian, Upper Cretaceous (‘mid-Cretaceous’).

#### Diagnosis

Head capsule with 12 stiff setae on tubercles (18 setae in *Postopsyllidium*); fore wing without pterostigma (tiny pterostigma, widening of ScP + RA present in *Postopsyllidium*); vein CuP not thickened distally (distinctly thickened distally in *Postopsyllidium*); profemur with a row of ventral (ventrolateral) setae (two rows in *Postopsyllidium*).

*Megalophthallidion burmapateron* Drohojowska et Szwedo, sp. nov.

LSID urn:lsid:zoobank.org:act:F3F971F4-AE04-4F41-98B0-9A0A04470625.

(Figs. 1A–F, 2A–I).

**Figure 1:**
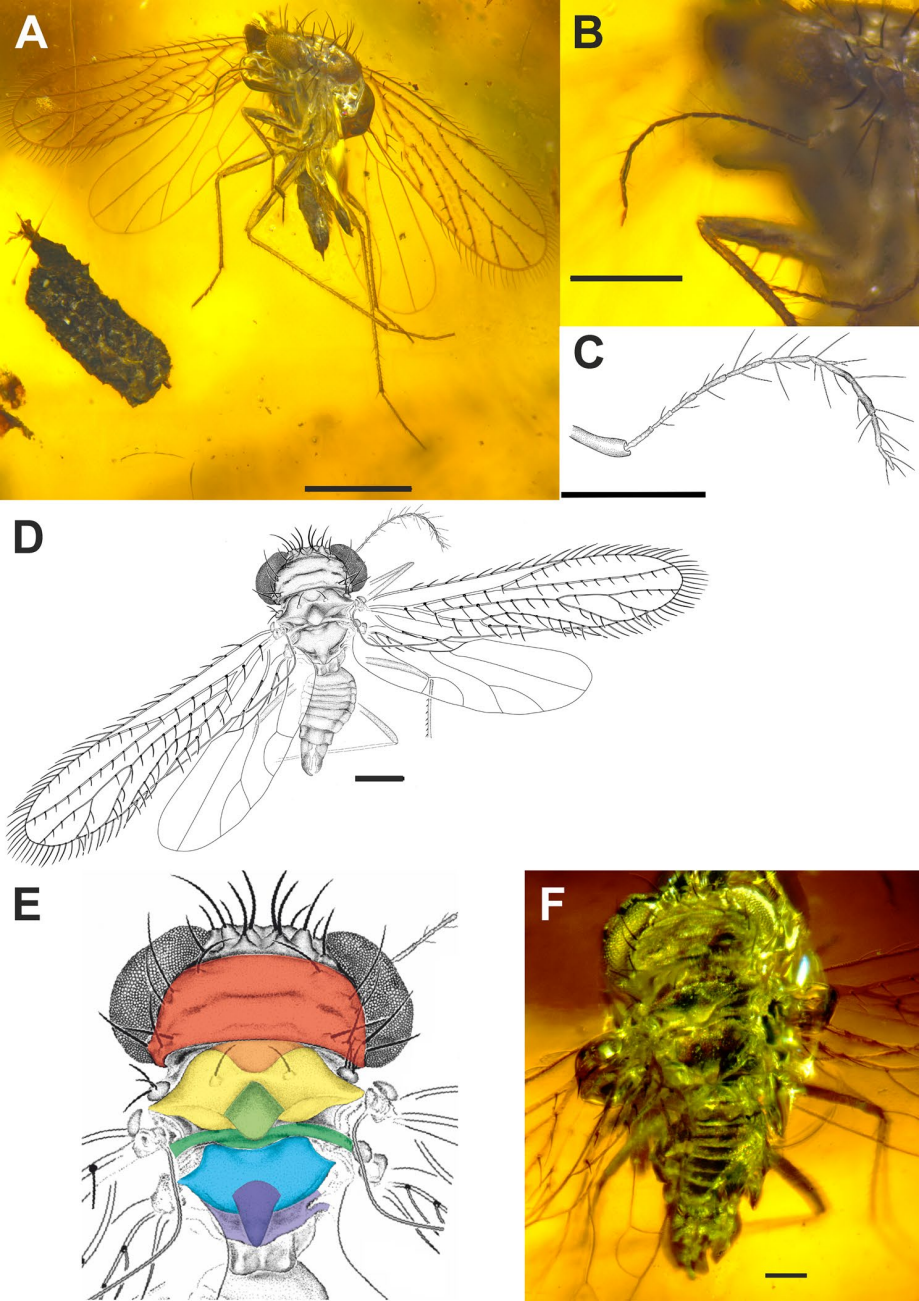
*Megalophthallidion burmapteron* gen. et sp. nov., holotype (MAIG 6687), imago. (**A**) Photo of body, ventral side; (**B**) photo of right antennae and (**C**) drawing of antenna; (**D**) drawing of body, dorsal side; (**E**) drawing of thorax structure with sclerites marked: red—pronotum; orange—mesopraescutum; yellow—mesoscutum; light green—mesoscutellum, dark green—mesopostnotum; light blue—metascutum; dark blue—metascutellum; violet—metapostnotum; (**F**) photo of thorax dorsal side. Scale bars: 0.5 mm (**A**), 0.2 mm (**B–D**), 0.1 mm (**F**).

**Figure 2:**
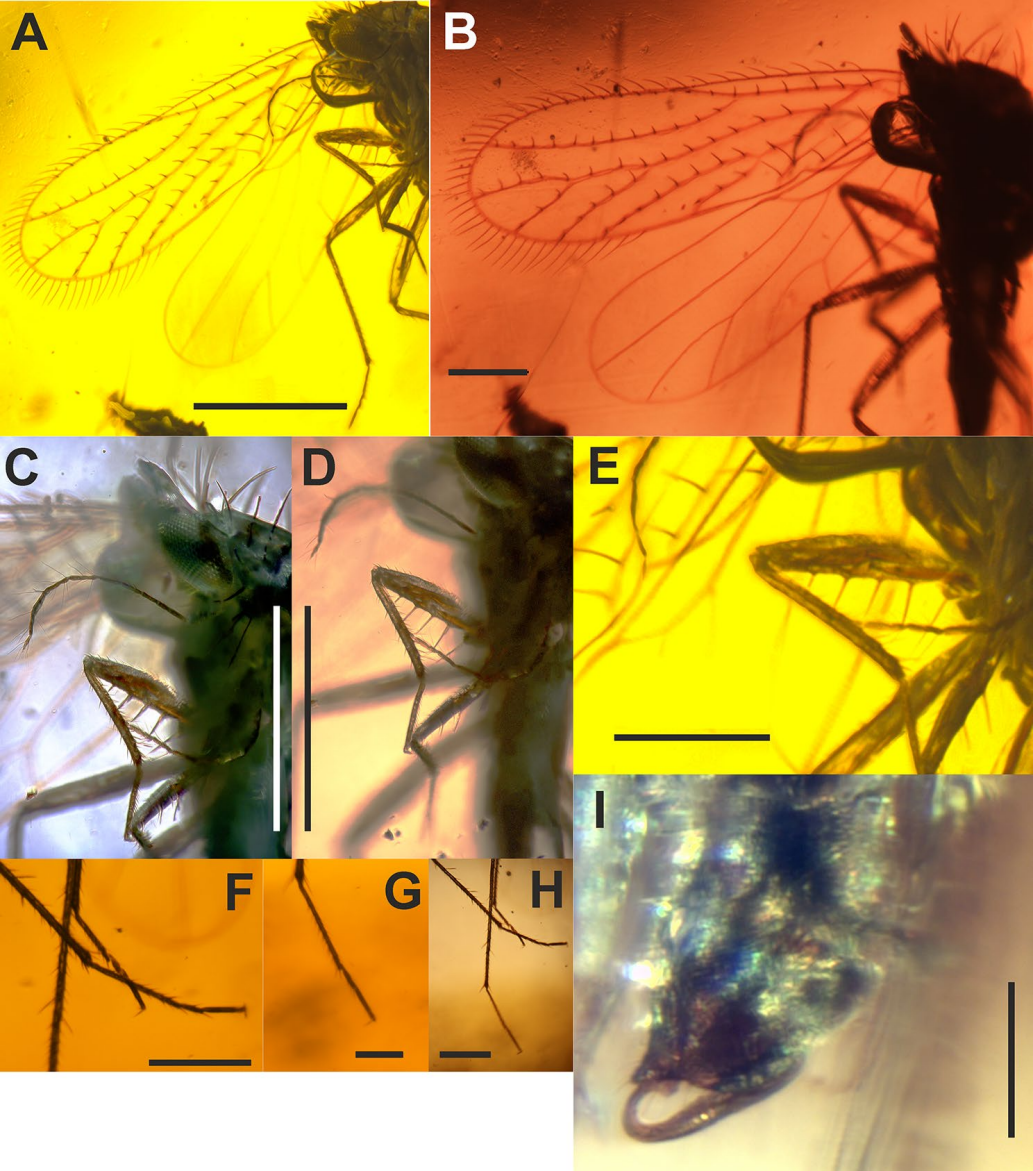
*Megalophthallidion burmapteron* gen. et sp. nov., holotype (MAIG 6687), imago. (**A**) Photo of right fore wing; (**B**) photo of right wings; (**C**) photo of antenna and proleg; (**D**) photo of proleg and mesoleg, and (**E**) photo of femur of proleg, and (**F**) photo of right metatarsus and left mesotarsus in the background, and (**G**) photo of right mesotarsus of mesoleg, and (**H**) Photo of tarsi; (**I**) photo of male genital block. Scale bars: 0.5 mm (**A–D**), 0.2 mm (**B,E,F,H**), 0.1 mm (**G,I**).

#### Material

Holotype, number MAIG 6687 (BUB 96), deposited in Museum of Amber Inclusions (MAIG), University of Gdańsk, Poland. Imago, a complete and well-preserved male. Piece of amber 8 × 6 × 3 mm, cut from larger lump, polished flat on both sides.

#### Type locality

Northern Myanmar: state of Kachin, Noije bum 2001 Summit Site amber mine in the Hukawng Valley, SW of Maingkhwan.

#### Type stratum

Lowermost Cenomanian, Upper Cretaceous (‘mid-Cretaceous’).

#### Diagnosis

As for the genus with the following additions: three ocelli distinct, antennomere IX the longest, about as long as pedicel, antennomeres III–VII and XI of similar length, antennomere XII the shortest, subconically tapered in apical portion. Paramere lobate, ventral margin with acute, small process, apical and dorsal margins rounded. Aedeagus geniculately bent at base, directed dorsally, tapered apicad.

#### Description

Male (Figs. 1A–F, 2A–I). Head with compound eyes distinctly wider than pronotum (Fig. 1D–F). Compound eyes subglobular, protruding laterally. Vertex short in midline, about 2.5 times as wide as posterior margin and as long in middle; trapezoidal, anterior margin slightly arched, lateral margins diverging posteriad, posterior margin shallowly arched, disc of vertex with distinct setae on large tubercles: four setae at posterior margin, two at anterior angles of compound eyes, two medial, over the median ocellus. Three ocelli present, median ocellus distinct, visible from above, lateral ocelli near anterior angles of compound eyes. Frons about as wide as long in midline, two rows of setae on tubercles, upper row at level of median ocellus, lower one, below half of compound eye height. Clypeus, elongate, triangular, in lower portion roof-like; two setae on tubercles near upper margin. Genae very narrow. Rostrum reaching slightly beyond mesocoxae, apical segment slightly shorter than subapical one, darker. Antennae bases placed at lower margin of compound eyes; antennal fovea elevated; scapus shorter than pedicel, cylindrical; pedicel cylindrical; antennomeres IIIrd–VIIth and XIth of similar length, VIIIth slightly longer than VIIth, as long as Xth antennomere, IXth the longest, XIIth the shortest, tapered apically; rhinaria absent.

Thorax (Fig. 1D–F): pronotum quadrangular, about as long as mesothorax; pronotum with anterior and posterior margins parallel, merely arcuate, disc with transverse groove in the median portion, lateral margins slightly arcuate, two distinct setae on tubercles in anterolateral angle, two setae on tubercles anterior margin at distance1/3 to median line, three distinct setae on tubercles in posterolateral angles. Mesopraescutum subtriangular, with apex widely rounded, about 0.4 times as wide as pronotum, about 0.4 times as long as wide, delicately separated from mesoscutum. Mesoscutum as wide as pronotum at widest point, distinctly narrowed medially, anterior angles rounded, anterolateral margin sigmoid, lateral angle acute, posterior angles wide, posterior margin V-shape incised, posterolateral areas of mesoscutum disc declivent posteriorly; disc with two setae on tubercles, at 1/3 of mesoscutum width. Mesoscutellum about as long as wide, diamond-shape, anterior and lateral angles acute, posterior angle rounded. Mesopostnotum in form of transverse band, slightly widened in median portion. Metascutum narrower than mesoscutum, anterior angles widely rounded, lateral angles acute, anterolateral margin concave, posterior margin arcuate, with deep median arcuate incision. The suture between metascutum and metascutellum weakly visible, metascutellum subtriangular, longer than wide at base.

Parapteron with three distinct setae.

Fore wing (Fig. 2A,B) membranous, narrow, elongate, about 3.5 times as long as wide, widest at 2/3 of length. Anterior margin merely arcuate, slightly bent at very base, anteroapical angle widely arcuate, apex rounded, posteroapical angle widely arcuate, tornus arcuate, claval margin straight, with incision between terminals of Pcu (claval apex) and A_1_. Stem ScP + R + MP + CuA slightly arcuate, very short stalk ScP + R + MP + CuA leaving basal cell, stem ScP + R oblique, straight, forked in basal half of fore wing length, branch ScP + RA, oblique, reaching anterior margin slightly distally of half of fore wing length, slightly distally of ending of CuA_2_ branch; branch RP slightly arcuate, a little more curved in basal section, reaching margin at anteroapical angle; stalk MP + CuA slightly shorter than basal cell; stem MP almost straight, forked in apical half of fore wing, at about 2/3 of fore wing length, with three terminals reaching margin between apex and posteroapical angle; stem CuA shorter than branches CuA_1_ and CuA_2_, about half as long as branch CuA_1_; claval vein CuP weak at base, not thickened distally; claval vein Pcu straight, claval vein A_1_ straight. Basal cell present, subtriangular, about twice as long as wide, basal veinlet *cua-cup* oblique, no other veinlets present; cell r (radial) very long, longer than half of fore wing length; cell m (medial) the shortest, shorter than cell cu (areola postica). Margins of fore wing with fringe of long setae, starting on costal margin near base of fore wing, ending at level of middle of cell cu; longitudinal veins with distinct, scarcely but evenly dispersed, movable setae; terminal section of CuP with two setae; costal margin with row of short, densely distributed setae, apical margin, tornus and claval margin with rows of scaly setae.

Hind wing (Fig. 2B) membranous, shorter than fore wing, 3.23 times as long as wide. Costal margin bent at base, then almost straight up to the level of ScP + RA end and wing coupling lobe, then straight to anteroapical angle, anteroapical angle widely arcuate, apex arcuate, posteroapical angle arcuate, tornus straight, claval margin merely arcuate, posteroclaval angle angulate; stem ScP + R + MP bent at base, then straight, stem ScP + R short, branch ScP + RA short, about as long as stem ScP + R, branch RP arcuate basally than straight, reaching apex; stem MP arcuate, forked slightly distad CuA_1_ terminus level, branch MP_1+2_ slightly arcuate, reaching margin at posteroapical angle, branch MP_3+4_ straight, reaching tornus; stem CuA slightly bent at base, then straight, forked slightly distad ScP + R forking, branch CuA_1_ arcuate, branch CuA_2_ short, straight, slightly oblique, reaching tornus; claval vein CuP weak, visible only at base, claval vein Pcu slightly arcuate; wing coupling apparatus (fold) with a few short setae.

Legs slender, relatively long, profemora armed (Fig. 2C–H). Procoxa as long as profemur, narrow, flattened. Protrochanter scaphoid, elongate, with long apical and subapical setae. Profemur flattened laterally, about as long as protibia, ventrally armed with four large setae on elevated plinths; dorsal margin with row of short, decumbent setae. Protibia narrow, rounded in cross section, covered with short setae, a few longer setae in distal portion. Protarsus—single, long tarsomere, plantar surface with row of semi-erect setae; tarsal claws long, straight, directed ventrally, no arolium nor empodium.

Mesocoxa elongate, narrow, slightly flattened. Mesotrochanter scaphoid. Mesofemur slender, flattened laterally, dorsal margin with short setae. Mesotibia subequal to mesofemur, slender, covered with setae, two apical setae slightly thicker and longer. Mesotarsus with three tarsomeres, basimesotarsomere the longest, shorter than cumulative length of mid- and apical mesotarsomere, plantar margins with setae, two apical setae slightly longer and thicker; midmesotarsomere the shortest, 1/3 of basimesotarsomere length, a few setae on plantar surface; apical tarsomere shorter than basimesotarsomere, twice as long as midmesotarsomere, plantar surface with a few, scarcely dispersed setae, tarsal claws long, narrow, directed ventrally, no arolium nor empodium.

Metacoxa conical, narrow. Metatrochanter scaphoid, elongate. Metafemur slender, laterally flattened, longer than mesofemur, dorsal margin with row of short setae. Metatibia, long, slender, 1.6 times as long as metafemur, with suberect setae of different size, two larger and longer and two shorter setae subapical setae. Metatarsus slightly less than half of metatibia length, with three tarsomeres, basimetatarsomere the longest, more than twice as long as apical metatarsomere, 1.5 times as long as combined length of mid- and apical metatarsomere, plantar surface with scarce decumbent setae; mid metatarsomere the shortest, 1/4 of basimetatarsomere length, plantar surface with a few setae, two apical ones slightly thicker; apical metatarsomere about 0.4 of basimetatarsomere length, with scarcely dispersed setae on along plantar surface; tarsal claws, long, slender, other pretarsal structures absent.

Abdomen (Fig. 1F) narrowly attached to thorax, tergite segment shorter, 2nd tergite distinctly longer, 3rd to 8th tergites of similar length; pygofer narrowing apicad, ventral margin strongly elongated posteriorly; anal tube short, directed posterodorsad, anal style shorter than anal tube. Paramere lobate, ventral margin with acute, small process, apical and dorsal margins rounded. Aedeagus (Fig. 2I) geniculately bent at base, directed dorsad, tapered apicad.

Female. Unknown.

*Megalophthallidion* sp. (5th instar nymph)

(Figs. 3A–D, 4A–F)

**Figure 3:**
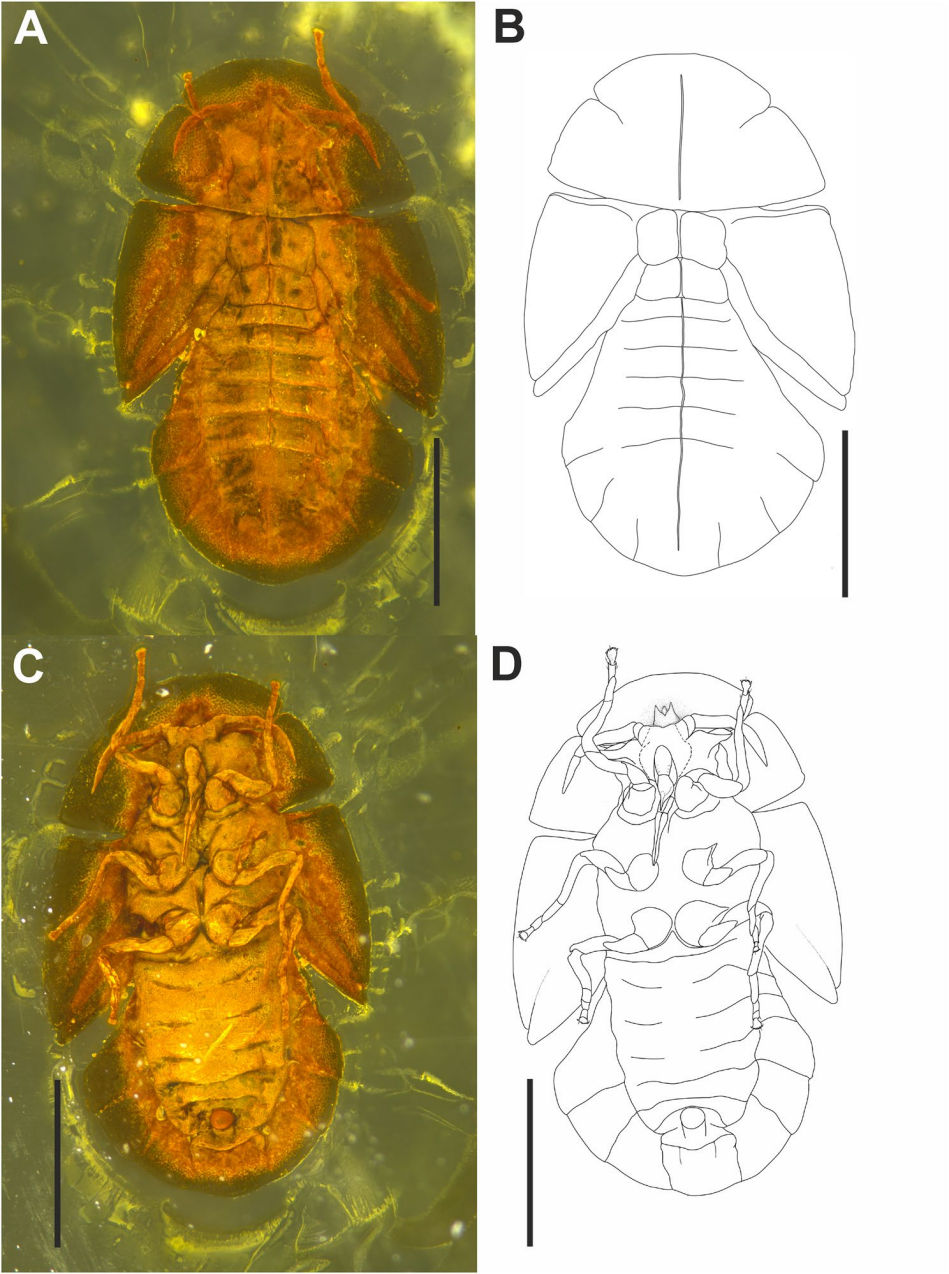
*Megalophthallidion* sp. (MAIG 6688), nymph. (**A**) Photo of body, dorsal side and (**B**) drawing of body dorsal side; (**C**) photo of body dorsal side and (**D**) drawing of body ventral side. Scale bars: 0.5 mm (**A–D**).

**Figure 4:**
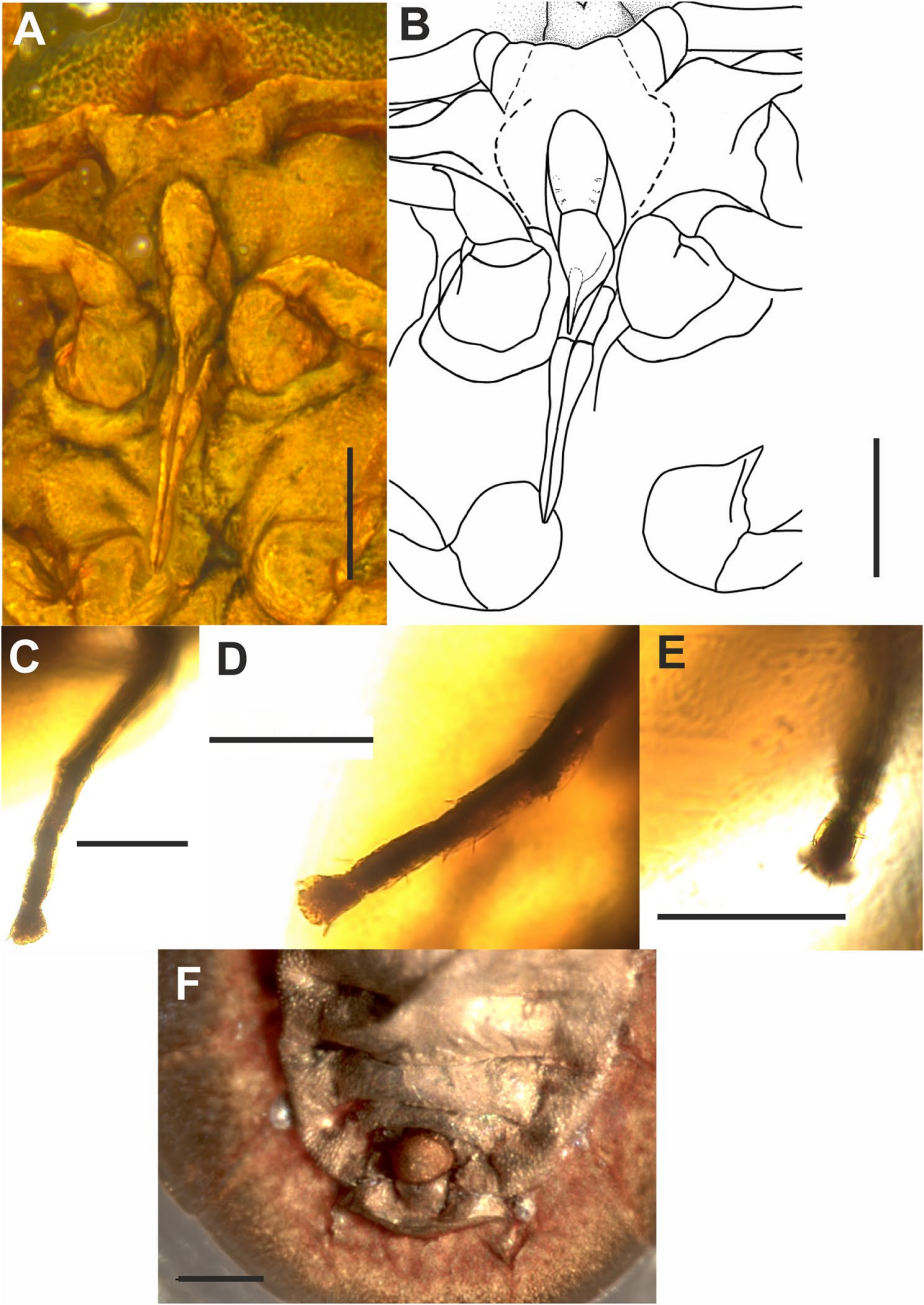
*Megalophthallidion* sp. (MAIG 6688), nymph. Photo of clypeus and (**B**) drawing of clypeus; (**C**) photo of proleg, and (**D**) photo of mesoleg, and (**E**) photo of metaleg; (**F**) photo of posterior part of abdomen ventral side. Scale bars: 0.1 mm (**A–F**).

#### Material

Nymph, 5th instar, MAIG 6688 (BUB 1799), deposited in Museum of Amber Inclusions (MAIG), University of Gdańsk, Poland. Piece of amber 13 × 6 × 2 mm, cut from larger lump, polished flat on one side, more convex on the other.

#### Diagnostic characters

The nymph of *Megalophthallidion* gen. nov. is similar in general body shape to the only known fossil protopsyllidioidean nymph described from Lower Cretaceous Lebanese amber—*Talaya batraba* Drohojowska et Szwedo, 2013. The nymph of *Talaya batraba* is 2nd or 3rd instar, therefore some features are difficult to compare with this last instar nymph of *Megalophthallidion* gen. nov. The morphological states observed in those two specimens are: head covered with strongly expanded disc and expanded disc of pronotum, however shapes and ratios of these structures differ; compound eyes on ventral side of head, shifted laterad (ommatidia on cones in *T. batraba*, while ventroposterior expansions are present in *Megalophthallidion* gen. nov.); compound eyes visible from above as short, stout cones in fissure between posterior margin of disc (hypertrophied vertex) and anterior margin of pronotum (compound eyes (?) are visible on dorsal side of Permian *Aleuronympha bibulla* Riek, 1974); in *Megalophthallidion* gen. nov. rostrum reached mesocoxa, while in *Talaya batraba* distinctly exceeds length of the body; abdomen with 9 segments; tergites of abdominal segments 5th–9th expanded posterolaterad in form of fan-like expansion; 9th abdominal segment short, placed ventral; anal tube short, cylindrical, epiproct (?) globular.

#### Description

Nymph, 5th instar (Figs. 3A–D, 4A–F). Body oval shaped, dorso-ventrally flattened, 1.5 times longer than wide with segmentation visible; on the ventral side slightly concave. Length of body *c*. 1.56 mm long, outline, in dorsal view, maximum width of body 0.94 mm; length of head and pronotum (cephaloprothorax) *c*. 0.46 mm in midline, width *c*. 0.83 mm; cumulative length of mesonotum + metanotum *c*. 0.25 mm; abdomen *c*. 0.8 mm long. Dorsal side (Fig. 3A,B) with distinct median line (ecdysial line), not reaching anterior or posterior margin of the body, the line distinctly roof-like in abdominal portion. Anterior margin of head (cephaloprothorax) disc arcuate, lateral angles rounded; anterior margin of pronotum arcuate, lateral margins arcuately diverging posteriad, posterior margin distinctly arcuate, anterior angles widely rounded, posterior angles acutely rounded, disc elevated, convex, lateral portions declivitous; the fissure between posterior margin of head disc and anterior margin of pronotum narrow, widened medially, with stalked compound eyes popping out.

Head partly separated from prothorax, wide in ventral view. Bases of antennae protruding anterolaterally, wide, anterior margin arcuate, with a small lump extending anteriorly connecting margin with vertex expansion. Suture separating anteclypeus and postclypeus visible in ventral aspect (Fig. 4A,B). Postclypeus about three times as long as wide, oval, slightly swollen, without any setae; weak traces of salivary pump muscle attachments visible. Anteclypeus about as long as postclypeus, widened in upper section below clypeal suture, convex, carinately elevated in lower section, with sides distinctly declivitous, clypellus long, carinately elevated. Lora (mandibulary plates) distinct, separated from anteclypeus by shallow suture, with upper angles at half of postclypeus length, lower angles at half of anteclypeus length, about as wide as half of postclypeus width. Maxillary plates narrow. Genal portion of head enlarged, medial portion arcuately convex; lateral sections narrowing laterally, terminally encircling bases of compound eyes. Antennae short (Fig. 3C,D), placed in front of genal portion. Antennal flagellum indistinctly subdivided into four segments. Rostrum (Fig. 4A,B) three-segmented, 0.2 mm long, with apex reaching apex of mesocoxae; apical segment about 2.5 times as long as subapical one.

No lateral sclerites on meso- and metathorax, only one *plus* one large medial sclerite on both meso- and metathorax. Mesothoracic and metathoracic wing pads distinct, wide, subtriangular, with posterior apices directed posteriorly; lateral portions of mesothoracic wing pads arcuate. Fore wing pad 0.6 mm long, with small, straight humeral lobe, forming a right angle, not protruding anteriorly. Mesothoracic tergites slightly larger than metathoracic segments (respectively *c*. 0.14 mm and *c*. 0.12 mm long in midline, 0.26 mm and 0.27 mm in lateral lines); mesothoracic tergum with distinct median elevation (low double crest with ecdysial line in between), slightly wider than long in midline, anterior margin arcuate, lateral margins straight, subparallel, posterior margin concave. Metathoracic wing pad apex slightly exceeding mesothoracic wing pad. Metathoracic tergum wider than long, slightly shorter than mesothoracic tergum, with distinct elevation in the middle.

Legs relatively long (Figs. 3C,D, 4C–E). Coxae of legs placed near the median axis of the body. Prolegs: procoxal pit with margins elevated, procoxa conical (*c*. 0.1 mm long), protrochanter scaphoid, about as long as procoxa, profemur *c*. 0.13 mm long, slightly flattened laterally, merely thickened, protibia longer than profemur, *c*. 0.23 mm long; tarsus shorter than protibia, basiprotarsomere about as long as apical protarsomere, the latter with distinct tarsal claws, and wide arolium. Mesoleg similar to proleg, mesocoxa conical (*c*. 0.1 mm long), mesotrochanter scaphoid, mesofemur (*c*. 0.13 mm) slightly flattened laterally, mesotibia slightly longer than mesofemur (*c*. 0.18 mm), mesotarsus slightly shorter than mesotibia, three-segmented, basimesotarsomere the longest (*c*. 0.07 mm), about as long as combined length of mid- and apical mesotarsomeres (*c*. 0.04 mm respectively), arolium wide, tarsal claws distinct. Metaleg: metacoxa conical (*c*. 0.1 mm), metatrochanter scaphoid, about as long as metacoxa (*c*. 0.12 mm). Metafemur (*c*. 0.17 mm) slightly more thickened than pro- and mesofemur, metatibia slightly longer (0.19 mm) than pro- and mesotibiae. Metatarsus three-segmented: basimetatarsomere about as long (0.08 mm) as combined length of mid- and apical metatarsomeres (0.04 mm respectively), arolium lobate, wide, tarsal claws distinct, widely spread.

Abdomen (Fig. 3A–D) 9-segmented, narrow at base, widening fan-shape posteriorly, 1st segment visible from above, segmentation visible, abdominal terga 5th–9th expanded posterolaterally. Tergites carinately elevated in the middle, separated by ecdysial line. 1st sternite visible in ventral view, sternites 2nd–4th fused medially, sternites 5th–9th separated; 9th abdominal segment short (Fig. 4F), placed ventrally, under tergal expansion; anal tube short, cylindrical, epiproct (?) globular.

## Discussion

The genus *Megalophthallidion* Drohojowska et Szwedo, gen. nov. described above is placed in Postopsyllidiidae based on the presence of stiff setae on head and thorax; antenna with 12 antennomeres; well developed pronotum; fore wing with marginal fringe and reduced pterostigma; branched stem of R; MP three-branched; well developed, separated claval veins A_1_ and A_2_; setae present on fore wings and hind wings bare; metatarsi with three tarsomeres, and aedeagus exposed^6^. It differs from *Postopsyllidium* by having a head capsule with 12 stiff setae on tubercles, while 18 setae are present in *Postopsyllidium*; its fore wing lacks a pterostigma, a structure which is vestigially present in *Postopsyllidium* as a widening of ScP + RA; vein CuP is not thickened distally, contrary to *Postopsyllidium* where it is distinctly thickened distally, and a profemur with a single row of ventral (ventrolateral) setae, while two rows are present in *Postopsyllidium*^6,16^.

Therefore, the Postopsyllididae encompasses the genus *Postopsyllidium* Grimaldi, 2003, with species *P. rebeccae* Grimaldi, 2003, *P. grimaldii* Hakim, Azar et Huang, 2019 in Hakim et al. 2019, *P. burmaticum* Hakim, Azar et Huang, 2019 in Hakim et al. 2019 (all Cenomanian amber from Kachin, Myanmar)^6,16^, *P. emilyae* Grimaldi, 2003 (amber from the Turonian Raritan Formationof New Jersey, U.S.A.)^16^, and the newly described genus and species *Megalophthallidion burmapateron* Drohojowska et Szwedo, gen. et sp. nov., from Cenomanian amber of Kachin, Myanmar.

Postopsyllidiidae presents a number of plesiomorphic characters undefined viz., venational pattern with single RP, three-branched MP, areola postica present, claval veins A_1_ and A_2_ present and distinct; antennae with 12 antennomeres, presence of 3 ocelli, 3-segmented tarsi (at least meso- and metatarsi). On the other hand, there are several apomorphic features distinguishing them from other Protopsyllidioidea, viz. loss of veinlet *rp-mp* on fore wing (the value of this character was disputed by Klimaszewski^17^); presence of setae on head, pronotum and mesonotum (however it could be taphonomic bias due to preservation in fossilized resin rather than as adpression fossils), setae on body are present also in Paraprotopsyllidiidae^7^; fringe of setae on the wings, which is also a character of Paraprotopsyllidiidae; setae along the veins are present also in Protopsyllidiidae, e.g., in the genus *Poljanka* Klimaszewski, 1995, but absent in Permopsyllidiidae^6,17–20^; lack of distinct pterostigmal area, present in Permopsyllidiidae and Protopsyllidiidae; male genital structures with aedeagus bent resembling the structure present in representatives of Psylloidea—however this feature could be the result of convergence^6^.

**Figure 5:**
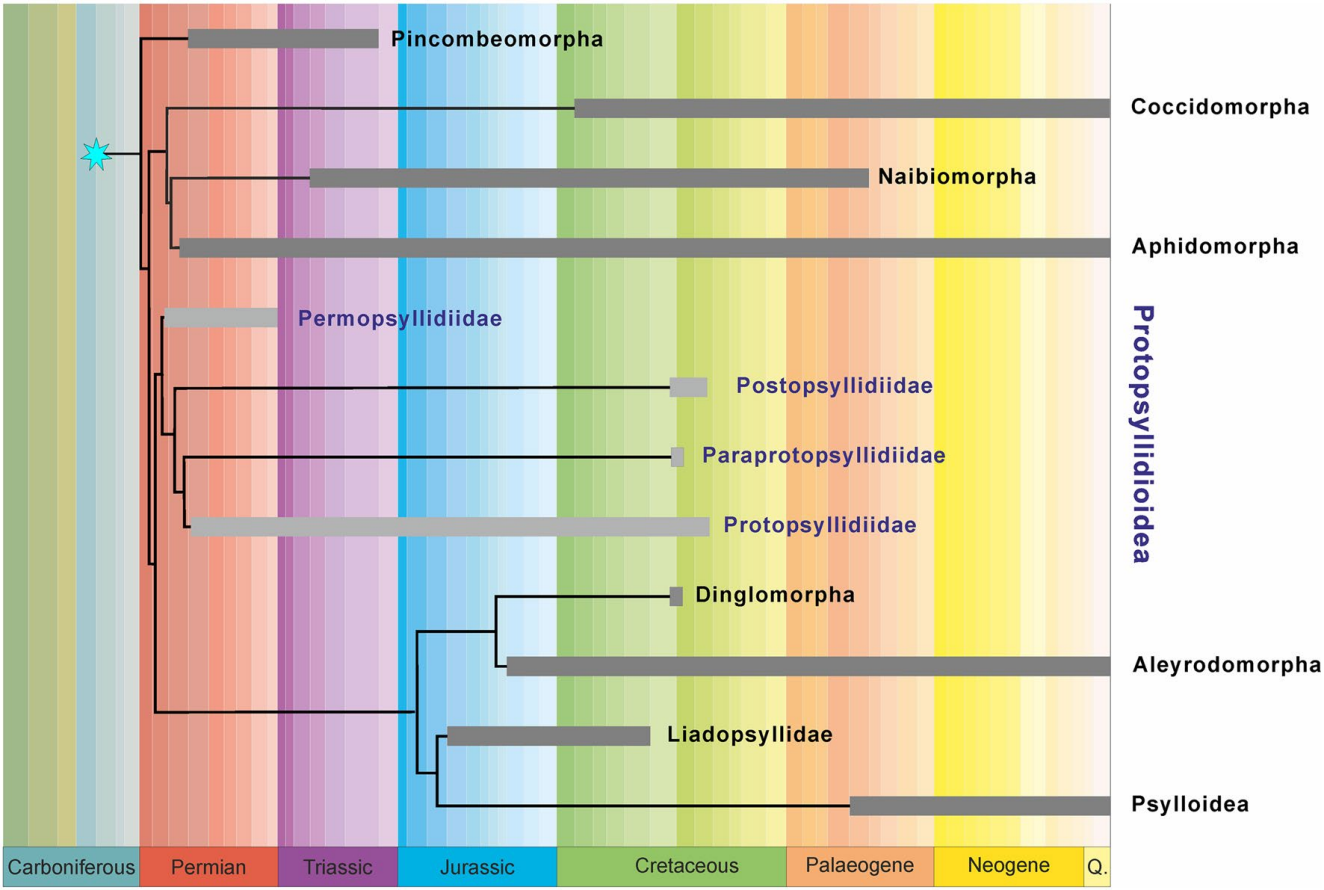
Tentative relationships of Protopsyllidioidea and other Sternorrhyncha (based on data and interpretations from Drohojowska et al.^14^ and Hakim et al.^7^; figure generated with use of CorelDraw 2020).

Nymphal features of Protopsyllidioidea are poorly known^6,21^. However, protopsyllidioids are known since the Sakmarian, early Permian^22^. The record of fossil protopsyllidioid nymphs comprises an unnamed lower Permian nymph (from the Kungurian Vryheid Fm., Middle Ecca Group, Hammanskraal, South Africa), one middle Permian species (*Permaleurodes rotundatus* Becker-Migdisova, 1959 from the Wordian Kazankovo-Markinskaya Formation of Kemerovo, Kuznetsk Basin, Russia), one upper Permian species (*Aleuronympha bibulla* Riek, 1974 from the Changhsingian Mooi River locality, South Africa, and an undescribed nymph from the Changhsingian Belmont Conglomerate Member, New South Wales, Australia)^23–26^. These fossils and their taxonomic positions have been previously discussed^21^ and their placement in Protopsyllidioidea (Fig. 5) is still debatable^6^. An undescribed nymph is mentioned from the Triassic (Carnian) Molteno Formation (Stormberg Group) of South Africa^27^. Shcherbakov^28^ reported the presence of protopsyllidioid nymphs in two Lower-Middle Jurassic localities: Iya in the Irkutsk Basin (Toarcian), and Novospasskoe in Buriatia (Toarcian/Alenian). The only nymph ascribed to Protopsyllidiidae is *Talaya batraba* Drohojowska, Szwedo et Azar, 2013 from Barremian Lebanese amber of Mdeyrij-Hammana, Lebanon^21^.

The character shared by all these nymphs and by the newly described specimen is exaggeration in size of the head and pronotum in dorsal view, with frontoclypeus, bases of compound eyes, bases of antennae, and frons hidden under the ‘helmet’ composed of the disc of vertex and laterally expanded pronotum. As this character has been present for a long time period, at least since the Permian, it seems to be very conservative. This could be related to the ancient, cryptic habits of these insects, as suggested by Shcherbakov and Popov^15^, who characterised the earliest hemipteran nymphs as dorsoventrally flattened, oval, cryptic, and unable to jump. Becker-Migdisova^29^ suggested that the Archescytinidae, with the ovipositor developed to various extents, laid eggs inside pteridospermous seed plants and early gymnosperms generative parts: strobiles or staminate cones, and the nymphs dwelt there until the ripe strobile dehisced. A similar pattern of larval habitats and habits was suggested for early Sternorrhyncha (e.g., protopsyllidioids), ancient Fulgoromorpha (e.g., *Knezouria unicus* Jell, 1993), Cicadomorpha, and Coleorrhyncha. The larval morphology reflects, to a certain extent, their biology: free-living larvae tend to be elongate with long limbs, pit-gall inducers are oval or circular and flattened dorsally, and closed-gall inhabitants are weakly sclerotized and ‘inflated’. Small, usually dorsoventrally depressed, oval, scone-like or biscuit-like morphs with short legs, small frontoclypeus and long rostrum might have fed on phloem of rather thick stems^15,30^. When and where the transition from generative organ to phloem feeding took place is unknown. It seems plausible that this phenomenon occurred very early in Sternorrhyncha evolution, probably at the boundary of the Carboniferous and Permian, due to global cooling^31^ and consequent changes in early gymnosperms morphology, with cones and cupules becoming less accessible (including their phloem bunches) and phloem bunches in twigs acting as an alternative resource^32,33^. Since then, a wide array of strategies seem to have been present among various lineages of Sternorrhyncha, resulting in the diversity of nymphal development known in extant species^1,9,10^. Protopsyllidioidea and Psylloidea seem to retain the most ‘conservative’ pattern of nymph structure, with the head weakly separated from thorax, antennae and legs with fewer segments than in imagines, legs not of jumping type, protopterons (wingpads) developed, terminal abdominal segments often fused, anal orifice in ventral, or apical position (dorsal in imagines); younger instars generally more sessile, performing intercellular stylet-penetration of plants tissues^34,35^. These characters are also present in the nymph of *Megalophthallidium* gen. nov. viz*.*, cryptic, flattened and with rounded body outline, head weakly separated from thorax, hidden under expanded lobes, short walking legs (non-jumping), anal orifice directed ventrally.

Interestingly, such a pattern of body structure is present in the slightly older, Early Cretaceous protopsyllidioid *Talaya batraba* Drohojowska et al., 2013 (Protopsyllidiidae), and in a younger fossil Aphalaridae psyllid from middle Eocene Baltic amber—*Eogyropsylla* Klimaszewski, 1993 described by Klimaszewski^36^. This evolutionary conservatism of psyllid nymphs is present also in extant representatives of the group^37,38^. Younger instars are more sessile and more strictly associated with their host plants, so this phenomenon could be also a reflection of their evolutionary histories. This could be the reason for the rarity of nymphal protopsyllidioids in the fossil record: their cryptic habitus, hiding and sessile feeding in crevices of bark rather than on leaves decreases their fossilization potential and only preservation in fossil resins provides a a glimpse of their morphology and disparity. It can be assumed that the Protopsyllidioidea were a rather successful group between the Permian and the Cretaceous, widely distributed and present in various habitats. Being rather conservative in their morphological disparity^6,7,16,18^ for most of their evolutionary history, they presented higher morphological diversity and adaptative rates during the mid- to Late Cretaceous, probably responding to challenges of the Cretaceous Terrestrial Revolution^39^. It is interesting to note that during this event, unrelated Psylloidea^13^ probably at least partly replaced protopsyllidioids in their ecological roles; Hodkinson^40^ hypothesized that the evolution of psyllids was driven by their host plants (mostly angiosperms), and he proposed the model of host plants as “the rafts transporting the insects down the river of evolutionary time”. Extant Psylloidea occupy a wide range of habitats, with *ca*. 4000 species of psyllids recognized^41^.

Regardless, there is no synthetic model of phylogenetic relationships for any of the sternorrhynchan groups, althoughr numerous competing proposals have been offered^13^. Most of the evolutionary history of divergence among sternorrhynchan species has yet to come to light^13^. The finding of new taxa of Postopsyllidiidae add new pieces to this complex jigsaw of Sternorrhyncha evolutionary history (Fig. 5).

## Materials and methods

The investigated specimens are inclusions in mid-Cretaceous amber from Kachin Province, northern Myanmar. The piece of amber with the nymph was cut, ground and polished and embedded in artificial resin for better visibility. A Nikon MZ1500, Nikon SMZ1270, Leica M205C stereoscopic microscopes, and a Nikon Microphot-FX equipped with a camera lucida, and changeable direct and transmitted light (Institute of Biology, Biotechnology and Environmental Protection, University of Silesia, Katowice) and Olympus SZX10 and Olympus BX42 (Laboratory of Evolutionary Entomology and Museum of Amber Inclusions, University of Gdańsk, Gdańsk) were used for the microscopic examination. The photographs were taken using the Nikon Microphot-FX with a Nikon Eclipse E 600 digital camera and Lucia® software, and Olympus SZX10 with Olympus EP50 camera under EPView v3.7.2) and adjusted using Adobe® Photoshop Elements 6.0.

Morphological terminology follows mostly Ossiannilsson^42^, Hollis^43^, Drohojowska^44^, the interpretation of veins after Hakim et al.^6,7^.

Both amber specimens with inclusions (MAIG 6687—imago, MAIG 6688—nymph) studied here come from the Cretaceous deposits in the Hukawng Valley of Kachin State, northern Myanmar (locality: Noije Bum; 26° 21′ 33.41′′ N, 96° 43′ 11.88′′ E; palaeocoordinates 12.4° N, 93.8° E; see Fig. 1 in Refs.^45–48^. Radiometric U–Pb zircon dating of the volcaniclastic matrix of the amber constrained a refined age of 98.79 ± 0.62 Ma (earliest Cenomanian)^49^. For more details on geology and palaeoenvironment of the area see Supplementary File.

The amber pieces were obtained by the original collectors before 2015. We began to analyze the fossils in early 2017 and finished all analyses at the end of 2021. To avoid any confusion and misunderstanding, all authors declare that the amber reported in this study was not involved in armed conflict and ethnic strife in Myanmar. The type specimen and nymph were purchased from a private collector and deposited in the publicly and permanently accessible Museum of Amber Inclusions (MAIG), in full compliance with the International Code of Zoological Nomenclature^50^, the indications of the International Palaeoentomological Society^51^ and policies proposed by Haug et al.^52^.

**Figure 6:**
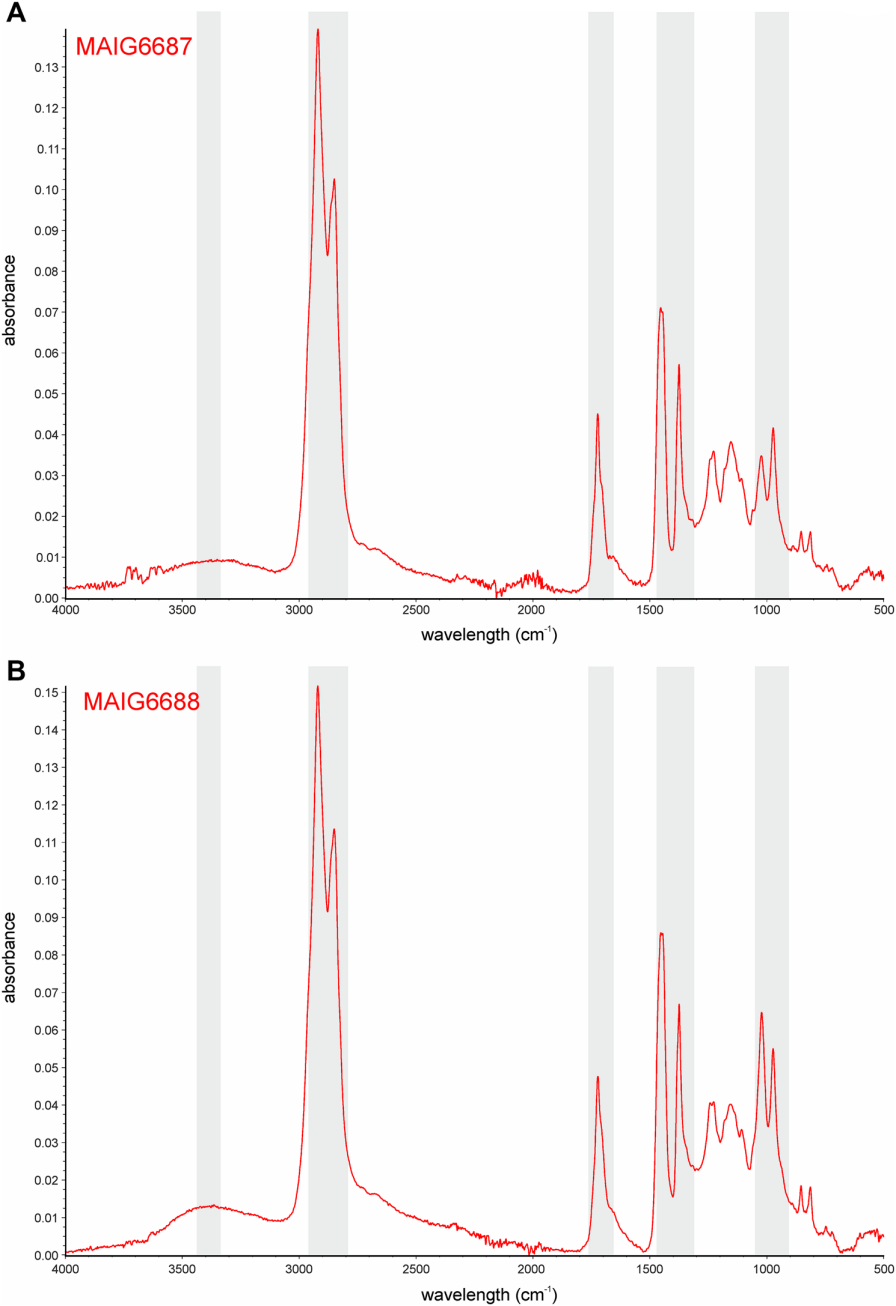
FT-IR spectra of the amber pieces. (**A**) Piece with inclusion of *Megalophthallidion burmapteron* gen. et sp. nov., holotype MAIG6687 and (**B**) Piece with inclusion of *Megalophthallidion* sp. nymph MAIG6688.

The specimens were provided with FT-IR spectra according to the procedure proposed by Szwedo and Stroiński^53^ to confirm the fossil resin provenance (Fig. 6A,B). The FT-IR spectra are registered as MAIG 6687FT-IR (imago) and MAIG 6688FT-IR (nymph) in the Museum of Amber Inclusions, University of Gdańsk, Gdańsk, Poland. The spectra were obtained in the Amber Experts company laboratory, Gdańsk, with Nicolet 380 spectrometer with ATR and baseline correction. The spectra received were analysed and compared with data presented in Refs.^54,55^, with fingerprint peaks confirming the origin of the amber pieces.

### Nomenclatural acts

The electronic edition of this article conforms to the requirements of the amended International Code of Zoological Nomenclature, and hence the new names contained herein are available under that Code from the electronic edition of this article. This published work and the nomenclatural acts it contains have been registered in ZooBank, the online registration system for the ICZN. The ZooBank LSIDs (Life Science Identifiers) can be resolved and the associated information viewed through any standard web browser by appending the LSID to the prefix ‘http://zoobank.org/’. The LSID for this publication is: urn:lsid:zoobank.org:pub:88FEFD52-A902-47B8-B890-53B27557D30E. The electronic edition of this work was published in a journal with an ISSN, and has been archived and is available from the following digital repositories: PubMed Central, CLOCKSS, Knowledge Base of the University of Gdańsk https://repozytorium.bg.ug.edu.pl/, and the Repository of the University of Silesia https://www.ciniba.edu.pl/repozytorium-re-bus-n.

## Supplementary Information

Supplementary Information.

## Data Availability

All data generated or analysed during this study are included in this published article and its Supplementary Information files.
