# Supplementary material for: Evolutionary implications of new Postopsyllidiidae from mid-Cretaceous amber from Myanmar and sternorrhynchan nymphal conservatism
Source: Sci Rep. 2022 Sep 30;12:16446. doi: 10.1038/s41598-022-20897-y (PMC9525616; doi:10.1038/s41598-022-20897-y)
Supplement: Supplementary file 1 — Supplementary Information. [file 41598_2022_20897_MOESM1_ESM.pdf]

Supplementary file to:

Evolutionary implications of new Postopsyllidiidae from mid-Cretaceous amber from Myanmar and sternorrhynchan nymphal conservatism

Jowita Drohojowska[1], Marzena Zmarzły[1], Jacek Szwedo[2,*]

[1] Institute of Biology, Biotechnology and Environmental Protection, University of Silesia, 9, Bankowa Street, PL40-007 Katowice, Poland; e-mail: jowita.drohojowska@us.edu.pl; marzena.zmarzly@us.edu.pl
[2] Laboratory of Evolutionary Entomology and Museum of Amber Inclusions, Department of Invertebrate Zoology and Parasitology, University of Gdańsk, 59, Wita Stwosza Street, PL80-308 Gdańsk, Poland; e-mail: jacek.szwedo@biol.ug.edu.pl

* Corresponding Author

JD ORCID https://orcid.org/0000-0002-7668-2242
MZ ORCID https://orcid.org/0000-0002-0631-6079
JS ORCID https://orcid.org/0000-0002-2796-9538

1. Geological setting

As the southward extension of the Tibetan orogeny, Myanmar lies south to the eastern Himalayan syntaxis, and is suggested to be subdivided into three major tectonic zones: the Indo-Burma range, the West Burma and Sibumasu Terranes[56-57]. The amber locality in this study lies in northern part of the West Burma Terrane[58], which was suggested to collide with the Sibumasu Terrane in early Cretaceous[59-60]. In mid to late Cretaceous, the West Burma Terrane was situated in the southern edge of the southeastern Asia, and the frontal area of the subduction of the Neo-Tethyan ocean to the Eurasian plate[56,57,59]. Humid tropical climate with volcanic activities thought to be dominated in this area, which can also be indicated from fossil records[45,46,61].

Burmese amber, the fossil resin referred as burmite[62,63], has become the most important source of amber inclusion in Cretaceous. There are more than 2000 species varying from invertebrates, vertebrates, protists, plants, and fungi have been reported from Burmese amber by now, indicating the enormous biodiversity and biotic importance of the Burmese amber biota[64]. The amber deposits are mainly outcrops as clastic sedimentary rocks, with thin limestone beds and abundant coaly and carbonaceous material. An estimated age of earliest Cenomanian (98.8 ± 0.6 Ma) for the amber deposit was proposed by[49] from radiometric U-Pb dating of zircons from the volcanoclastic matrix. This age is younger than the age based on fossil ammonite[65], pholadid bivalves inclusion and palynological study[61,66-68]. Xing & Qiu[69] also proposed an older zircon U-Pb dating age (ca. 110 Ma) from the newly discovered Hkamti mine south to the Hukawng basin, and indicated the importance of distinguishing amber sources from different mining regions. Detailed geological surveys are still needed to constrain various amber bearing layers in the Cretaceous strata in the northern Myanmar.

The Burma Terrane was part of a Trans-Tethyan island arc and stood at a near-equatorial southern latitude at about 100 Ma. The area of Kachin amber formation and deposition in the mid-Cretaceous times is considered to be an island or archipelago[70-74,] suggesting island endemism for the Kachin amber biota. A warm, humid, nearshore marine setting with high species diversity has been proposed for the amber locality[65,46]. The origin of the resin remains unclear, but Cupressaceae gymnosperms, very likely *Metasequoia* Hu & W.C. Cheng, 1948 or related taxa, were the trees exuding the resin at the time of burmite formation[75] as well as trees of the families Araucariaceae and Taxodiaceae[61,68,76]. The Cretaceous has often been described as a period of "warm and equable" climate[77], with the average global temperature near to 18°C. However, more recent refined work shows that climates during the 79 Myr of the Cretaceous were not quite so unvarying as originally thought[78-80]. Larger meridional heat transport by atmospheric and/or oceanic circulation, shaping the mid-Cretaceous "supergreenhouse" period[81] inferred the area of Kachin amber formation and deposition in the mid-Cretaceous times. Combination of topography and climatic challenges could be responsible for magnification of evolutionary rate of insects in this area, resulting in their adaptation and fast diversification.

2. Taxonomic treatments

To avoid a lengthy list of references dealing with scientific names and being in concordance with ICZN[50] Article 51.1 and Recommendations 22A.1 and 51A, and following Consortium of European Taxonomic Facilities (CETAF) best practices in electronic publishing in taxonomy[82] here we are listing all taxonomic names with their authors and references to the papers in which the taxa mentioned in the main text have been established. We are following the convention that taxon authority is treated as part of the name, not a citation, but relevant paper introducing the name of a taxon must be listed. The names of taxa are presented here in alphabetical order.

Aleuronympha bibulla Riek, 1974[83]
Aphalaridae Löw, 1879[84]
Archescytinidae Tillyard, 1926[85]
Cicadomorpha Evans, 1946[86]
Coleorrhyncha Myers et China, 1929[87]
Eogyropsylla Klimaszewski, 1993[88]
Fulgoromorpha Evans, 1946[86]
Hemiptera Linnaeus, 1758[89]
Knezouria unicus Jell, 1993[90]
Paraprotopsyllidiidae Hakim, Azar, Szwedo, Drohojowska et Huang, 2021[7]
Permopsyllidiidae Becker-Migdisova, 1985[18]
Permaleurodes rotundatus Becker-Migdisova, 1959[24]
Poljanka Klimaszewski, 1995[17]
Postopsyllidiidae Hakim, Azar et Huang, 2019[6]
Postopsyllidium Grimaldi, 2003[16]
Postopsyllidium burmaticum Hakim, Azar et Huang, 2019 in Hakim *et al*. 2019[6]
Postopsyllidium emilyae Grimaldi, 2003[16]

Postopsyllidium grimaldii Hakim, Azar et Huang, 2019 in Hakim *et al*. 2019[6]
Postopsyllidium rebeccae Grimaldi, 2003[16]
Protopsyllidiidae Carpenter, 1931[91]
Protopsyllidioidea Carpenter, 1931[91]
Sternorrhyncha Amyot et Audinet-Serville, 1843[92]
Talaya batraba Drohojowska, Szwedo et Azar, 2013[21]

Additional references
(not listed in the main text)

56. Li, J.X., *et al.* Mesozoic-Cenozoic tectonic evolution and metallogeny in Myanmar: Evidence from zircon/cassiterite U-Pb and molybdenite Re-Os geochronology. *Ore Geol. Rev.* **102**, 829–845; 10.1016/j.oregeorev.2018.10.009 (2018).
57. Metcalfe, I. Gondwana dispersion and Asian accretion: Tectonic and palaeogeographic evolution of eastern Tethys. *J. Asian Earth Sci.* **66**, 1–33; 10.1016/j.jseaes.2012.12.020 (2013).
58. Broly, P., Maillet, S. & Ross, A.J. The first terrestrial isopod (Crustacea: Isopoda: Oniscidea) from Cretaceous Burmese amber of Myanmar. *Cret. Res.* **55**, 220–228; 10.1016/j.cretres.2015.02.012 (2015).
59. Gardiner, N.J., *et al.* The tectonic and metallogenic framework of Myanmar: A Tethyan mineral system. *Ore Geol. Rev.* **79**, 26–45; 10.1016/j.oregeorev.2016.04.024 (2016).
60. Zaw, K., Swe, W., Barber, A.J., Crow, M.J. & Nwe, Y.Y. Introduction to the geology of Myanmar. Myanmar: Geology, Resources and Tectonics. *Geol. Soc. Mem.* **48**, 1–17; 10.1144/M48.1 (2017).
61. Cruickshank, R.D. & Ko, K. Geology of an amber locality in the Hukawng Valley, Northern Myanmar. *J. Asian Earth Sci.* **21**, 441–455; 10.1016/S1367-9120(02)00044-5 (2003).
62. Helm, O. On a new, fossil, amber-like resin occurring in Burma. *Rec. Geol. Surv. Ind.* **25** (4), 180–181 (1892).
63. Helm, O. Further note on Burmite, a new amber-like fossil resin from Upper Burma. *Rec. Geol. Surv. Ind.* **26** (2), 61–64 (1893).
64. Ross, A.J. Supplement to the Burmese (Myanmar) amber checklist and bibliography, 2021. *Palaeoentomology* **5** (1), 27–45; 10.11646/palaeoentomology.5.1.4 (2022).
65. Yu, T. T., *et al.* An ammonite trapped in Burmese amber. *Proc. Natl. Acad. Sci.* **116**, 11345–11350; 10.1073/pnas.1821292116 (2019).
66. Wright, C.W, Calloman, J.H, & Howarth, M.K. *Treatise on intertebrate paleontology, Part L. Mollusca 4 (revised). Volume 4: Cretaceous Ammonoidea*, xx+362 pp. (The Geological Society of America and the University of Kansas, Boulder, Colorado and Lawrence, Kansas, 1996).
67. Mao, Y. *et al*. Various amber ground marine animals in Burmese amber with discussions on its age. *Palaeoentomology* **1** (1), 91–103; 10.11646/palaeoentomology.1.1.11 (2018).
68. Smith, R.D.A. & Ross, A.J. Amberground pholadid bivalve borings and inclusions in Burmese amber: implications for proximity of resin-producing forests to brackish

waters, and the age of the amber. *Earth Env. Sci. T.R. So.* **107** (2-3), 239–247; 10.1017/S1755691017000287 (2018).

69. Xing, L., Qiu, L. Zircon U-Pb age constraints on the mid-Cretaceous Hkamti amber biota in northern Myanmar. *Palaeogeogr. Palaeoclimatol. Palaeoecol.* **558**, 109960, 1–11; 10.1016/j.palaeo.2020.109960 (2020).
70. Rasnitsyn, A.P. & Öhm-Kühnle, C. Three new female *Aptenoperissus* from mid-Cretaceous Burmese amber (Hymenoptera, Stephanoidea, Aptenoperissidae): Unexpected diversity of paradoxical wasps suggests insular features of source biome. *Cret. Res.* **91**, 168–175; 10.1016/j.cretres.2018.06.004 (2018).
71. Xing, X. L. *et al.* A mid-Cretaceous embryonic-to-neonate snake in amber from Myanmar. *Sci. Adv.* **4** (eaat5042), 1–8; 10.1126/sciadv.aat50 42 (2018).
72. Xing, L., *et al.* Hummingbird-sized dinosaur from the Cretaceous period of Myanmar. *Nature* **579**, 245–249; 10.1038/s41586-020-2068-4 (2020).
73. Westerweel, J. *et al.* Burma Terrane part of the Trans-Tethyan arc during collision with India according to palaeomagnetic data. *Nat. Geosci.* **12**, 863–868; 10.1038/s41561-019-0443-2 (2019).
74. Morley, C.K., Naing, T.T., Searle, M. & Robinson, S.A. Structural and tectonic development of the Indo-Burma ranges. *Earth-Sci. Rev.* **200**, 102992; 10.1016/j.earscirev.2019.102992 (2020)
75. Grimaldi, D.A. & Ross, A.J. Extraordinary Lagerstätten in amber, with particular reference to Cretaceous of Burma in *Terrestrial conservation Lagerstätten: windows into the evolution of life on land* (ed. Fraser, N. & Sues, H.-D.) 287–342 (Dunedin Academic Press, Edinburgh 2017).
76. Poinar, G. Jr., Lambert, J.B. & Wu, Y. Araucarian source of fossiliferous Burmese amber: spectroscopic and anatomical evidence. *J. Bot. Res. Inst. Texas* **1**, 449–455 (2007).
77. Hay, W.W. Toward understanding Cretaceous climate – An updated review. *Sci. China Earth Sci.* **60**, 5–19; 10.1007/s11430-016-0095-9 (2016).
78. Holz, M. Mesozoic paleogeography and paleoclimates – a discussion of the diverse greenhouse and hothouse conditions of an alien world. *J. S. Am. Earth Sci.* **61**, 91–107; 10.1016/j.jsames.2015.01.001 (2015).
79. Huber, B.T., Macleod, K. G., Watkins, D. K., & Coffin, M. F. The rise and fall of the Cretaceous Hot Greenhouse climate. *Glob. Planet. Change* **167**, 1–23; 10.1016/j.gloplacha.2018.04.004 (2018).
80. Vickers, M.L., Price, G.D., Jerrett, R.M., Sutton, P., Watkinson, M. P. & Meriel FitzPatrick, M. The duration and magnitude of Cretaceous cool events: Evidence from the northern high latitudes. *Geol. Soc. Am. Bull.* **131**, 1979–1994; 10.1130/B35074.1 (2019).
81. Hasegawa, H., *et al.* Drastic shrinking of the Hadley circulation during the mid-Cretaceous Supergreenhouse. *Clim. Past.* **8**, 1323–1337; 10.5194/cp-8-1323-2012 (2012).
82. Bénichou L., Gérard I., Laureys É. & Price M.J. Consortium of European Taxonomic Facilities (CETAF) best practices in electronic publishing in taxonomy. *Eur. J. Taxon.* **475**: 1–37; 10.5852/ejt.2018.475 (2018).

83. Riek E.F. An unusual immature insect from the Upper Permian of Natal. *Ann. Natal Mus.* **22** (1), 271–274 (1974b).
84. Löw, F. Zur Systematik der Psylloden. *Verh. d. k. k. Zool.-Bot. Ges.* **28**, 586–610; biostor.org/reference/61412 (1879).
85. Tillyard, R.J. Kansas Permian Insects, Part 9: The Order Hemiptera. *Am. J. Sci.* Series 5 **11** (65), 381–395; 10.2475/ajs.s5-11.65.381 (1926).
86. Evans, J.W. A natural classification of leaf-hoppers (Jassoidea, Homoptera) Part 1. External morphology and systematic position. *Trans. R. Entomol. Soc. Lond.* **96**, 47–60; 10.1111/j.1365-2311.1946.tb00442.x (1946).
87. Myers, J.G. & China, W.E. XXXVII.—The systematic position of the Peloridiidæ as elucidated by a further study of the external anatomy of *Hemiodœcus leai*, China (Hemiptera, Peloridiidæ). *Ann. Mag. Nat. Hist.* **3** (15), 282–294; 10.1080/00222932908672971 (1929).
88. Klimaszewski, S.M. New species of Eocene psyllids representing the tribe Palaeopsylloidini Becker-Migdisova (Homoptera, Psylloidea). *Acta Biol. Siles.* **22** (39), 9–18 (1993).
89. Linnaeus, C. *Systema naturae per regna tria naturae, secundum classes, ordines, genera, species, cum characteribus, differentiis, synonymis, locis. Tomus I. Editio decima, reformata*. (Laurentius Salvius, Holmia, 1758).
90. Jell, P.A. Late Triassic homopterous nymph from Dinmore, Ipswich Basin. *Mem. Queensl. Mus.* **33**, 360 (1993).
91. Carpenter, F.M. The Lower Permian insects of Kansas. Part 4. The order Hemiptera, and additions to the Paleodictyoptera and Protohymenoptera. *Am. J. Sci.* **5** (22), 113–130 (1931).
92. Amyot, C.J.-B. & Audinet-Serville, J. G. *Deuxième partie. Homoptères. Homoptera Latr. Histoire naturelle des insects. Hemiptères* 1–676; 10.5962/bhl.title.8471 (Librairie encyclopédique de Roret, Paris, 1843).
